# Impact of the COVID-19 pandemic on the epidemiology of traffic accidents: a cross-sectional study

**DOI:** 10.1590/0100-6991e-20223364-en

**Published:** 2022-11-23

**Authors:** ANGEL ADRIANY DA SILVA, GABRIELA REDIVO STRÖHER, HELOÍSA MORO TEIXEIRA, MARIA VICTÓRIA GUTIERREZ CORDEIRO, MARCIA OLANDOSKI, LUIZ CARLOS VON-BAHTEN

**Affiliations:** 1 - Hospital Universitário Cajuru, Liga Acadêmica do Trauma (LATHUC) - Curitiba - PR - Brasil; 2 - Pontifícia Universidade Católica do Paraná, Escola de Medicina e Ciências da Vida, Departamento de Bioestatística - Curitiba - PR - Brasil; 3 - Universidade Federal do Paraná, Departamento de Clínica Cirúrgica - Curitiba - PR - Brasil; 4 - Pontifícia Universidade Católica do Paraná, Escola de Medicina e Ciências da Vida, Departamento de Clínica Cirúrgica - Curitiba - PR - Brasil

**Keywords:** Epidemiology, Traumatology, Accidents, Traffic, COVID-19, Epidemiologia, Traumatologia, Acidentes de Trânsito, COVID-19

## Abstract

**Objective:**

to assess the epidemiological profile of traffic accident victims in the setting of the Coronavirus Disease 2019 (COVID-19) pandemic and analyze the admissions throughout the different levels of restriction (flags), as well as compare the results with the pre-pandemic period.

**Methods:**

a cross-sectional study was performed, with probability sampling, in a trauma center in Brazil. Medical records of patients involved in traffic accidents from June 2020 to May 2021 were evaluated. Aside from epidemiological characteristics, variables such as the current flag, the trauma mechanism, the resulting injuries, and the Revised Trauma Score (RTS) were also considered. Data were compared between three different flag periods and the proportion of consultations during the pandemic was compared with that from pre-pandemic time (December 2016 to February 2018).

**Results:**

it was observed that 62.2% of the patients were victims of motorcycle accidents, 77.5% were male, and the mean age was 33 ± 12.4 years. The mean and median RTS were 7.5 and 7.8, respectively. Statistical difference was stated when comparing the number of visits per day between the yellow and red flags (p=0.001) and orange and red flags (p=0.016). A significantly lower number of consultations for traffic accidents was observed in the pandemic when compared to the pre-pandemic period.

**Conclusions:**

the epidemiological profile of the study consisted mostly of young men who were victims of motorcycle accidents. There was a lower incidence of admissions during red flag periods and a lower proportion of consultations throughout the survey when compared to the pre-pandemic period.

## RESUMO

**Introdução:**

avaliar o perfil epidemiológico das vítimas de acidentes de trânsito no contexto da pandemia da doença do coronavírus 2019 (COVID-19), analisar os atendimentos entre os níveis de restrição implementados (bandeiras) e comparar os dados com o período pré-pandêmico.

**Métodos:**

trata-se de um estudo transversal, com amostragem probabilística, realizado em um hospital de trauma no sul do Brasil utilizando prontuários de pacientes vítimas de acidente de trânsito entre junho de 2020 a maio de 2021. Além das variáveis epidemiológicas, coletou-se a bandeira vigente, o mecanismo de trauma, as lesões resultantes e o Revised Trauma Score (RTS). Os dados foram comparados entre as três bandeiras e a proporção de atendimentos do período pandêmico foi comparada com a do período pré-pandêmico (dezembro de 2016 a fevereiro de 2018).

**Resultados:**

observou-se que 62,2% dos pacientes foram vítimas de acidentes com motocicletas, 77,5% da amostra era do sexo masculino e que a média etária foi de 33 ± 12,4 anos. A média e mediana do RTS foram 7,5 e 7,8, respectivamente. Houve diferença significativa ao comparar o número de atendimentos ao dia entre as bandeiras amarela e vermelha (p=0,001) e laranja e vermelha (p=0,016). Constatou-se um número significativamente menor de atendimentos por acidentes de trânsito no período pandêmico quando comparado com o período pré-pandêmico.

**Conclusões:**

o perfil epidemiológico do estudo foi composto em sua maioria por homens jovens vítimas de acidentes com motocicleta. Houve menor incidência de admissões na bandeira vermelha e menor proporção de atendimentos no período da pesquisa quando comparado ao pré-pandêmico.

## INTRODUCTION

Traffic accidents are one of the main external causes of trauma in the world. Annually, they cause the death of more than 1.3 million people and generate disabilities in more than 30 million, mainly in the age group between 15 and 29 years^1^ ^,^ ^2^. In Brazil, according to the Federal Highway Police Department, around 150,000 people were involved in traffic accidents on Brazilian highways in 2020, with 5,287 deaths recorded^3^. This extremely significant morbidity and mortality generates high costs for health systems, with socioeconomic impacts to the population^1^ ^,^ ^4^.

The continuous survey of epidemiological data regarding traffic accidents is of utmost importance, especially in different public health scenarios. In addition to assessing the prevalence and change in morbidity and mortality from trauma, it makes it possible to predict which injuries will be more frequent in periods of emergency and instability, directing medical care^5^ ^,^ ^6^.

In parallel, in March 2020, the disease caused by SARS-CoV-2 (COVID-19) was considered a pandemic by the World Health Organization (WHO). As it has respiratory characteristics of high transmissibility, it generated an overload on health systems^8^. So far, more than 338 million cases have been confirmed worldwide, despite the high rate of underreporting^7, 9,10^.

To stop the advance of this disease, non-pharmacological measures were instituted worldwide. The encouragement of social distancing and isolation, the suspension of services considered non-essential, and the implementation of hygiene protocols, such as the use of masks and hand washing, were some of them^11^. Following these recommendations, the city of Curitiba (Brazil) began in June 2020 the application of the Health and Social Responsibility Protocol, which divided the restriction measures into flags, according to the situation of the pandemic. The yellow flag represents a constant state of alert, emphasizing the application of contagion precautionary measures. The orange flag signals a moderate risk situation, restricting the operation of services that manage the agglomeration of people and implementing the curfew. Finally, the red flag signals a high-risk situation, limiting the movement of people and allowing only essential services to operate^12^.

As a result of the implementation of measures to combat COVID-19, several countries registered a decrease in the movement of people, a process that contributed to the change in the profile of trauma care in several hospitals around the world^13^ ^-^ ^17^.

Considering the current scenario and the importance of constantly updating epidemiological data regarding trauma, the objective of this study is to evaluate the epidemiological profile of victims of traffic accidents admitted to the Emergency Room of a reference hospital in Curitiba during the COVID-19 pandemic, and to analyze whether there was a difference in attendance between levels of restriction implemented and compare the results with those of the period prior to the pandemic.

## METHODS

This is a cross-sectional study with probability sampling carried out in a university hospital in southern Brazil. We collected data from medical records of patients admitted to the emergency room between June 2020 and May 2021. We included patients aged 18 years and over admitted via the emergency room through a medical rescue service or by direct search. We excluded incomplete medical records or those that evolved to death.

The sample size calculation was based on data from the study in the period prior to the pandemic (December 2016 to February 2018)^18^ and on the initial sample of 100 successive consultations that took place during the pandemic. To detect a significant difference between the distributions on the classifications of the trauma mechanism (traffic accidents, assaults, and falls), when comparing the pre-pandemic and pandemic periods, 833 patients would be needed, considering the level 5% significance and 80% test power. The sampling of the medical records took place using the GraphPad software. Alternate days were selected within the study period and, on each of them, a fifth of the medical records of patients who were victims of traffic accidents were drawn. If the exclusion criteria were met, a new draw was carried out.

The information in these documents was collected using a Google form and the variables considered were date, day of the week, whether or not it is a holiday, the restriction flag during which the event took place, victim’s age and sex, transport to the hospital by the public rescue service, highway concessionaire rescue service, direct search, or helicopter, trauma mechanism, and presence of protective equipment at the time of the trauma (helmet and three-point seat belt). Initial in-hospital care variables were Glasgow Coma Scale (GCS), systolic blood pressure (SBP), respiratory rate (RR), presence of extremity trauma, open fracture and/or traumatic brain injury, suicide attempt, and hospitalization.

As for medical transport services, SIATE (Integrated Trauma Care Service), created in 1990 in Curitiba, is integrated with the Fire Department and provides assistance to trauma victims. On the other hand, the SAMU (Mobile Emergency Care Service), created in 1995 and existing throughout Brazil, attends to all types of medical emergencies, including trauma^18^.

We used the variables GCS, SBP and RR to calculate the RTS (Revised Trauma Score), a physiological score that assesses the morbidity and mortality of polytraumatized patients. The score varies from 0 to 8, allowing fractions, and the higher its final value, the better the prognosis and probability of patient survival^19^.

Regarding statistical analysis, the results of age were described by mean, standard deviation, and minimum and maximum, and the categorical variables, by frequency and percentage. The association between mechanism and type of trauma were adjusted by logistic regression models. The significance of the variables was evaluated by the Wald test and the estimated association measure was the Odds Ratio with respective 95% confidence intervals. We used the nonparametric Kruskal-Wallis test to compare the RTS score between the groups established by the three restriction flags, and later performed a two-by-two analysis with Bonferroni-corrected significance. In addition, we used the Chi-square test to analyze the association of each of the categorical variables related to traffic accidents with the 3 levels of restriction in relation to the distributions on the variable classifications. We compared the restriction levels two-by-two regarding the incidence of attendances due to car accidents. Finally, we used the Chi-square test to compare the proportion of visits for car accidents and others (aggression and falls) in the period of this study with visits performed before the pandemic in the same hospital for such causes^18^. Values of p<0.05 indicated statistical significance. For the analyzes that showed statistical significance in the chi-square test, we analyzed the residuals, considering that there is an association between the variables in the cells that have an adjusted standardized residual value greater than 1.96. Data were organized in a Microsoft Excel® spreadsheet and analyzed using the IBM SPSS Statistics software, v.20.0. Armonk, NY: IBM Corp. Strategies to correct missing data were not adopted.

The project was approved by the Ethics in Research Committee (CEP) and has as identification the number of the Certificate of Ethical Appreciation (CAAE) 40014320.2.0000.0020.

## RESULTS

We included 426 patients treated for traffic accidents in the study, among them 62.2% for accidents involving motorcycles. The mean age was 33 ± 12.4 years (18-75 years), 50% of the patients were between 18 and 29 years old, and 77.5% were male (Table 1).

**Table 1:** Statistical analysis of study variables.

Variable	Classification	n=426 n(%)
Age	18-29	213 (50%)
	30-39	101 (23.7%)
	40-49	63 (14.8%)
	50-59	30 (7%)
	60-69	16 (3.8%)
	≥70	3 (0,7%)
Sex	Female	96 (22.5%)
	Male	330 (77.5%)
Day of week	Monday	56 (13.1%)
	Tuesday	54 (12.7%)
	Wednesday	63 (14.8%)
	Thursday	53 (12.4%)
	Friday	74 (17.4%)
	Saturday	65 (15.3%)
	Sunday	61 (14.3%)
Holiday	No	409 (96%)
	Yes	17 (4%)
Medical Rescue Service	SIATE	322 (75.6%)
	SAMU	73 (17.1%)
Variable	Classification	n=426 n(%)
	Highway concessionaire rescue service	25 (5.9%)
	Direct search	4 (0.9%)
	Helicopter	2 (0.5%)
Trauma Mechanism	Motorcycle collision	210 (49.3%)
	Motorcycle fall	55 (12.9%)
	Automobile collision	54 (12.7%)
	Run over	45 (10.6%)
	Bicycle collision	29 (6.8%)
	Bicycle fall	16 (3.8%)
	Rollover	14 (3.3%)
	Truck collision	3 (0.7%)
Grouped trauma mechanism	Motorcycle collision/fall	265 (62.2%)
	Bicycle collision/fall	45 (10.6%)
	Run over	45 (10.6%)
	Car, truck collision or rollover	71 (16.7%)

Regarding day of week, 47% of the events took place between Friday, Saturday, and Sunday (n=200) and only 4% took place on holidays. In addition, 75.6% of patients were transported to the hospital by SIATE and 17.1% by SAMU (Table 1).

Regarding the use of protective equipment, 68.5% (n=37) of patients involved in car accidents reported using a three-point seat belt and 84.2% (n=261) of victims of accidents involving motorcycles/bicycles used helmet. In two cases (0.5%) there was a suicide attempt.

The RTS score had a mean of 7.5 and a median of 7.8, with a minimum value of 2.6 and a maximum of 7.8. As for injuries, 39% of patients (n=166) suffered trauma to the extremities, 9.2% (n=39) had open fractures, and 8.7% (n=37) suffered traumatic brain injury (TBI). In addition, 35.7% (n=152) were hospitalized to resolve their clinical condition. When comparing the mechanisms of trauma involving motorcycles/bicycles and automobiles, there was a greater chance of trauma to the extremities (Odds Ratio 2.5, 95% CI 1.39 4.53, p=0.002), open fracture (Odds Ratio 4.39, 95% CI 1.03 18.70, p=0.045), and higher probability of hospitalization (Odds Ratio 3.05, 95% CI 1.61 5.81, p<0.001) in the motorcycle/bicycle group (Table 2).

**Table 2:** Comparison of injuries according to grouped trauma mechanisms.

Variable	Classification	Trauma Mechanisms		p***
		MB* (n=310)	CA** (n=71)	
Extremity Trauma	No	173 (55.8%)	54 (76.1%)	
	Yes	137 (44.2%)	17 (23.9%)	0.002
Open fracture	No	275 (88.7%)	69 (97.2%)	
	Yes	35 (11.3%)	2 (2.8%)	0.045
Traumatic brain injury	No	284 (91.6%)	65 (91.5%)	
	Yes	26 (8.4%)	6 (8.5%)	0.986
Patient will be hospitalized	No	184 (59.4%)	58 (81.7%)	
	Yes	126 (40.7%)	13 (18.3%)	<0.001

*MB: motorcycle + bicycle accidents group. **CA: car accidents + rollover group. ***Logistic regression model and Wald test, p<0.05.

Regarding the restriction periods, when comparing the number of visits per day in relation to traffic accidents, there was a significant difference between the yellow and red flags (p=0.001) and between the orange and red ones (p=0.016) (Table 3).

**Table 3:** Number of traffic accident attendances per day between restriction periods (classification by flags).

Flag	Number of days	Number of events	Events per day
Yellow	116	1155	10.0
Orange	224	2098	9.4
Red	26	205	7.9

There was no significant difference between the yellow and orange flags (p=0.096) (Table 3).

There was also a statistically significant difference in the comparison of RTS scores in the three periods (Table 4). When comparing the other categorical variables in this context (Table 5), there was no significant difference (p>0.05).

**Table 4:** Analysis of the RTS score from each restriction level (flags).

Flag	RTS score				p*	p**	
	n	Mean	Median	Minimum	Maximum		
Yellow	130	7.4	7.8	4.1	7.8		Yellow x Orange: p=0.006
Orange	267	7.5	7.8	2.6	7.8	<0.001	Yellow x Red: p=0.013
Red	29	7.1	7.1	6.4	7.8		Orange x Red: p<0.001

*Nonparametric Kruskal-Wallis test for the comparison between the three groups, p<0.05. **Significance adjusted by Bonferroni correction for the comparison of the groups two by two, p<0.05.

**Table 5:** Variables referring to each restriction level.

Variable	Classification	Flag			p*
		Yellow	Orange	Red	
Sex	Female	29 (22.3%)	61 (22.9%)	6 (20.7%)	
	Male	101 (77.7%)	206 (77.2%)	23 (79.3%)	0.963
Grouped trauma mechanism	Motorcycle collision/fall	81 (62.3%)	166 (62.2%)	18 (62.1%)	
	Bicycle collision/fall	11 (8.5%)	31 (11.6%)	3 (10.3%)	
	Run over	13 (10%)	28 (10.5%)	4 (13.8%)	
	Car, truck collision or rollover	25 (19.2%)	42 (15.7%)	4 (13.8%)	0.925
Extremity Trauma	No	76 (58.5%)	163 (61.1%)	21 (72.4%)	
	Yes	54 (41.5%)	104 (39%)	8 (27.6%)	0.379
Open fracture	No	112 (86.2%)	247 (92.5%)	28 (96.6%)	
	Yes	18 (13.9%)	20 (7.5%)	1 (3.5%)	0.065
Variable	Classification	Flag			p*
		Yellow	Orange	Red	
Traumatic brain injury	No	121 (93.1%)	242 (90.6%)	26 (89.7%)	
	Yes	9 (6.9%)	25 (9.4%)	3 (10.3%)	0.682
Suicide attempt	No	129 (99.2%)	266 (99.6%)	29 (100%)	
	Yes	1 (0.8%)	1 (0.4%)	0 (0%)	-
Hospitalization	No	78 (60%)	179 (67%)	17 (58.6%)	
	Yes	52 (40%)	88 (33%)	12 (41.4%)	0.312

*Chi-square test, p<0.05; (-) It was not possible to perform the Chi-square test given the low expected frequency of the data (more than 20% of the cells have an expected frequency lower than 1).

Finally, when comparing the proportion of trauma mechanisms in the pre-pandemic periods and during the COVID-19 pandemic in the same hospital, there was a significant difference (p=0.031). When analyzing standardized residuals, there was a significantly higher proportion of traffic accident attendances in the pre-pandemic period (Table 6).

**Table 6:** Comparison of the proportions of attendances of the three trauma mechanisms between the pre-pandemic and pandemic periods.

Trauma Mechanism	Pre-pandemic^18^ (2016-2018)	COVID-19 pandemic (2020-2021)	p*
	n (%) - [Residuals#]	n (%) - [Residuals#]
Traffic accidents	658 (53.1%) - [2.49]	426 (47.7%) - [-2.49]	
Assaults	229 (18.5%) - [-0.44]	172 (19.2%) - [0.44]	0.031
Falls	352 (28.4%) - [-2.33]	296 (33.1%) - [2.33]	
Total	1239 (100%)	894 (100%)	

*Chi-square test significance, p<0.05; #Adjusted standardized residuals, which for each cell results from: (observed frequency - expected frequency)^2^ ÷ expected frequency. Values greater than 2 indicate significant association/difference between variables. Positive residuals indicate a direct relationship, while negative ones indicate an inverse relationship.

## DISCUSSION

The COVID-19 pandemic has had major repercussions around the world, including changes in urban mobility patterns and traffic accidents. We observed that the epidemiological profile of traffic accidents during the analyzed period was that of young men who were victims of accidents involving motorcycles, which strongly coincides with what was found in the literature^20^ ^-^ ^22^. This predominance can be explained by the exponential growth in demand for home delivery services during the pandemic, in view of social distancing measures^21^ ^,^ ^23^. The need for fast deliveries may have generated long and stressful working hours, inducing fatigue and distractions in traffic and, consequently, an increased risk of negligence and involvement in accidents^24^. In addition, the prevalence of males in the 18-29 age group can usually be explained by the social and cultural behavior of this population^21^. Greater tendency to drive at high speed, disrespecting traffic rules, and drinking alcohol or using drugs before driving are reckless behaviors that make young people the main victims of traffic accidents^21^ ^,^ ^25^. As for the analysis of events according to the days of the week, we observed that the majority took place between Friday and Sunday, which coincided with what was found in another Brazilian study^21^.

As for the medical rescue service, SIATE provided pre-hospital care for 75.6% of patients, which is close to the percentage of the period prior to 2020 for traffic accidents (72.45%)^18^. This fact can be related to SIATE being an emergency trauma care service, while SAMU, despite also rescuing traumatized victims and supporting the former, is predominantly responsible for clinical emergencies^18^. Another probable reason for this result is the relative unawareness of the population of the difference between the two services and, who therefore make the wrong connection at the time of emergency. These factors, added to the study hospital being a reference for trauma in the city of Curitiba, justify the predominance of visits by SIATE, followed by SAMU.

The high percentage of helmet use by victims of motorcycle accidents in this study proved to be more promising when compared to other previous articles in the literature, in which adherence was close to 30%^25^. Furthermore, these findings may be linked to the low percentage of patients who had TBI (8.7%), since not using helmets makes them more susceptible and vulnerable to this injury^21^. In general, motorcyclists and cyclists are more likely to experience multiple trauma due to increased exposure during impact with other vehicles or bulkheads. This justifies the greater probability of these patients being hospitalized and the significant association between accidents by these mechanisms and trauma to the extremities, as shown in this study.

In the analysis of the variables within the restriction levels (flags), we found a significant difference in the value of the RTS score during the three flags. Despite this, the means and medians presented values above 7, a result similar to that of a South Korean study in 2020^26^, evidencing a good probability of patient survival. This result and the absence of a significant difference in the comparison between the other variables in these three periods may be associated with the effectiveness of the restriction measures, reducing the population’s exposure to high-energy mechanisms and their injuries.

When comparing the incidence of traffic accidents in the three periods, there was no difference between the yellow and orange flags. During the pandemic, the City Hall decreed the change of flags as there were variations in the number of COVID-19 cases and in the occupation of health services. The reduction of these rates, associated with the advance of vaccination in the city from 2021, made some rules of the yellow and orange flags to be relaxed^27^, which may explain the result found. In addition, there was a longer duration of these two levels of restriction during the analyzed period. A study^28^ showed that a prolonged period of restrictions decreases the probability of compliance by the population. Unemployment, debt, and the stress caused by social isolation are some of the reasons for this behavior^28^.

There was a lower incidence of traffic accidents during the red flag when compared to the others. This result is similar to that of studies carried out in a more restrictive scenario in this period^17^ ^,^ ^29^. More severe measures, associated with the worsening of the pandemic, encourage their compliance, given the concern for the greater chance of exposure to SARS-CoV-2.

During the pandemic period, there was a significantly lower proportion of attendances for traffic accidents compared with the pre-pandemic period (n=426 vs n=658) in the same hospital^18^, largely coinciding with other studies^20^ ^,^ ^21^ ^,^ ^30^ ^-^ ^33^. The restriction measures instituted by the City Hall, the closing of non-essential services, and the introduction of the home office system by several companies restricted the movement of vehicles and pedestrians in the city, which corroborates the result of this study.

With the advancement of vaccination, improvement in the rates of contagion of the virus, and long periods of restrictions, it is possible that the population has relaxed and failed to comply with the rules, which may have been a limitation of this study. It is known that the longer they last, the less likely people are to comply with them.

## CONCLUSION

The epidemiological profile of trauma victims in the study hospital during the COVID-19 pandemic was young men involved in motorcycle accidents. There was a lower incidence of attendances for traffic accidents during the red flag in relation to the other levels of restriction, and a lower proportion of attendances when compared with the pre-pandemic period.
